# A new mouse model for retinal degeneration due to *Fam161a* deficiency

**DOI:** 10.1038/s41598-021-81414-1

**Published:** 2021-01-21

**Authors:** Avigail Beryozkin, Chen Matsevich, Alexey Obolensky, Corinne Kostic, Yvan Arsenijevic, Uwe Wolfrum, Eyal Banin, Dror Sharon

**Affiliations:** 1 Department of Ophthalmology, Hadassah Medical Center, Faculty of Medicine, The Hebrew University of Jerusalem, 91120 Jerusalem, Israel; 2 Department of Ophthalmology, Jules-Gonin Eye Hospital, University of Lausanne, 1004 Lausanne, Switzerland; 3 Institute for Molecular Physiology, Johannes Gutenberg University, 55128 Mainz, Germany

**Keywords:** Genetics, Molecular biology, Developmental biology, Cell growth, Ciliogenesis, Disease model

## Abstract

*FAM161A* mutations are the most common cause of inherited retinal degenerations in Israel. We generated a knockout (KO) mouse model, *Fam161a*^*tm1b/tm1b*^, lacking the major exon #3 which was replaced by a construct that include LacZ under the expression of the *Fam161a* promoter. LacZ staining was evident in ganglion cells, inner and outer nuclear layers and inner and outer-segments of photoreceptors in KO mice. No immunofluorescence staining of Fam161a was evident in the KO retina. Visual acuity and electroretinographic (ERG) responses showed a gradual decrease between the ages of 1 and 8 months. Optical coherence tomography (OCT) showed thinning of the whole retina. Hypoautofluorescence and hyperautofluorescence pigments was observed in retinas of older mice. Histological analysis revealed a progressive degeneration of photoreceptors along time and high-resolution transmission electron microscopy (TEM) analysis showed that photoreceptor outer segment disks were disorganized in a perpendicular orientation and outer segment base was wider and shorter than in WT mice. Molecular degenerative markers, such as microglia and CALPAIN-2, appear already in a 1-month old KO retina. These results indicate that a homozygous *Fam161a* frameshift mutation affects retinal function and causes retinal degeneration. This model will be used for gene therapy treatment in the future.

## Introduction

Retinitis pigmentosa [RP (MIM #268000)] is the most prevalent hereditary degeneration of the retina in humans, with a prevalence of 1:4500 (in Europe and USA)^1–4^, and 1:2100 in the vicinity of Jerusalem^5^. RP is genetically and clinically heterogeneous, patients in early stages report reduced night vision and narrowing of the visual field, which are caused by death of rod photoreceptors. Subsequently, cone photoreceptors degenerate, leading to loss of central vision, decrease in visual acuity and eventually total blindness. Patients with RP usually have severely diminished or completely absent a-waves on electroretinographic (ERG) testing and bone spicule-like pigmentations (BSPs), attenuation of retinal vessels and a waxy pallor of the optic disc on funduscopy.

Currently, mutations in more than 60 genes were linked to non-syndromic RP, 41 of them were reported to cause autosomal recessive disease (RetNet, https://sph.uth.edu/retnet/), including *FAM161A*, which was initially identified in Israeli, Palestinian, Indian, and European patients^6,7^.

Clinical analysis of 100 Israeli patients with biallelic *FAM161A* mutations revealed some unique features including relatively slow degeneration of the photoreceptor layer up to the age of ~ 30 and good preservation of the photoreceptor cells in the macula until late age^8^, features which will enable therapeutic intervention (gene therapy, for example) in the future. While the phenotype of *FAM161A* patients is well described, the exact function of the encoded protein/s and the pathological mechanisms of the disease remain unknown.

Retinal analysis of Fam161a revealed its localization to the base of the photoreceptor connecting cilium, the synaptic regions of the outer and inner plexiform layers and the ganglion cells^9,10^ and takes a part of the cytoskeleton fraction of the mouse photoreceptor sensory cilium complex^11,12^. Different studies showed that Fam161a is part of microtubule-organizing centers, has a role in stabilization of existing microtubules^9^ and in the assembly of the primary cilium in cell cultures^10^. FAM161A binds directly to microtubules and its presence cause an increase in the acetylation of α-tubulin and stabilization of microtubules^9^. FAM161A contains a single highly conserved domain, termed UPF0564, located at the C-terminal region that is predicted to mediate microtubule association, interaction with other UPF0564 containing proteins and allows it to bind to more than 50 other ciliary proteins, such as LCA5 and CEP290^9,10^. In addition, FAM161A has been reported to be a member of the Golgi-centrosomal interactome that is involved in Golgi maintenance, intracellular transport and centrosome organization and to follow the centrosome through all stages of mitosis during cell cycle^13^. Thus, this protein is not limited to ciliary tasks only, but also has some additional cellular functions.

Animal models for retinal degeneration usually provide insights into pathological mechanism of disease progression and assist in designing therapeutic strategies. Up to date several animal models and particular mouse models for retinal diseases have been reported which occurred spontaneously and other models which have induced mutations in known retinal genes^14,15^. Some of these animal models served for gene therapy^15–19^. A mouse model for *Fam161a* deficiency, created using the GeneTrap system, has previously been reported^20^. Although retinal degeneration was evident in this model, the mutation site was considered “leaky” because it weakly express a truncated form of *Fam161a* in the inner segment of retinal photoreceptor cells, a situation that does not reflect the mutations identified in human patients that are considered null^6,8,21–33^. This mouse model therefore is less pertinent to study complete FAM161A loss.

In this study we report the generation and characterization of *Fam161a*^*tm1b*^^*/tm1b*^—a KO mouse model for *Fam161a*, which exhibits retinal degeneration with autosomal recessive inheritance. We show that mutated mice carry an out-of-frame deletion of the main exon (#3) in *Fam161a* producing a short RNA transcript that does not encode the major functional protein domain and is likely to be true null. The disease in our animal model mimics the disease in humans, suggesting that it may be a useful research tool for further disease characterization and gene augmentation therapy.

## Results

### Generation of ***Fam161a***^tm1b/Tm1b^ mice

**Figure 1:**
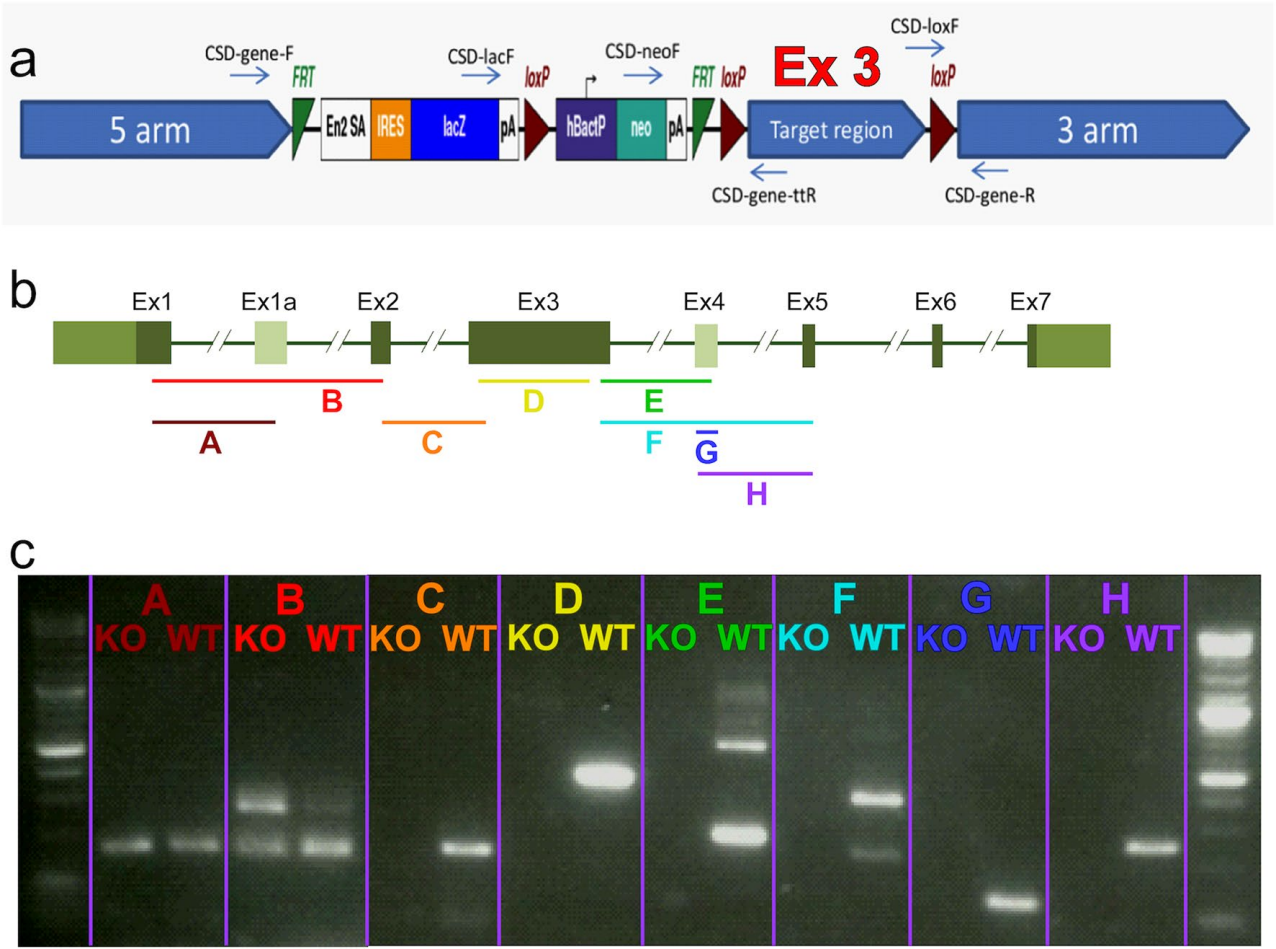
*Fam161a* knock out cassette and RNA expression in the mouse retina. (**a**) Schematic representation of the cassette (edited based on https://www.komp.org/ProductSheet.php?cloneID=635102) inserted into the mouse genome, targeting exon #3 of *Fam161a* (as provided by the KOMP company). (**b**) Schematic representation of the *Fam161a* structure. Dark green are exons that appear in all the transcripts, light green are alternative exons that appear in part of the transcripts. Color fragments represent the predicted RNA products in a specific PCR reaction. (**c**) RT-PCR products of different fragments of the *Fam161a* RNA in WT and *Fam161a*^tm1b/tm1b^ mice. Each color represents a different fragment which fits the color on the top panel.

We engineered a homozygous mouse line with targeted deletion of exon number 3 in *Fam161a* (*Fam161a*^tm1b/tm1b^) using a selection cassette that contains FRT and LoxP sites (Fig. 1a), which defines the area around exon #3 from position 23019523 at chromosome 11 to position 23021927 (exon 3 is located between positions 23019999–23021133). The LoxP sites were activated in order to remove the flanked area, and created the following deletion: NC_000077.6 (NM_028672.3): g.23019523_23021927del, as verified using Sanger sequencing of PCR products.

### ***Fam161a*** expression in*** Fam161a***^tm1b/Tm1b^ mouse retinas

Aiming to study the mutation effect at the RNA level, we performed RT-PCR analysis on *Fam161a*^tm1b/tm1b^ and WT retina at the age of 1 month using eight pairs of primers (Fig. 1b, fragments A-H), covering exons 1–5. We were able to identify RNA expression of the two first fragments (A and B, covering exons 1, 1a, and 2) in WT and *Fam161a*^tm1b/tm1b^ mice approximately at the same level (Fig. 1c). In fragment B, a band that corresponds to the isoform that contains a novel exon #1a (Supplementary Note) is more intense in mutant RNA comparing to WT. Starting from fragment C and downstream, we were not able to identify any RNA expression in mutated mice, though the expression in WT mice was evident (Fig. 1c). The sequence of all bands was verified by Sanger sequencing.

**Figure 2:**
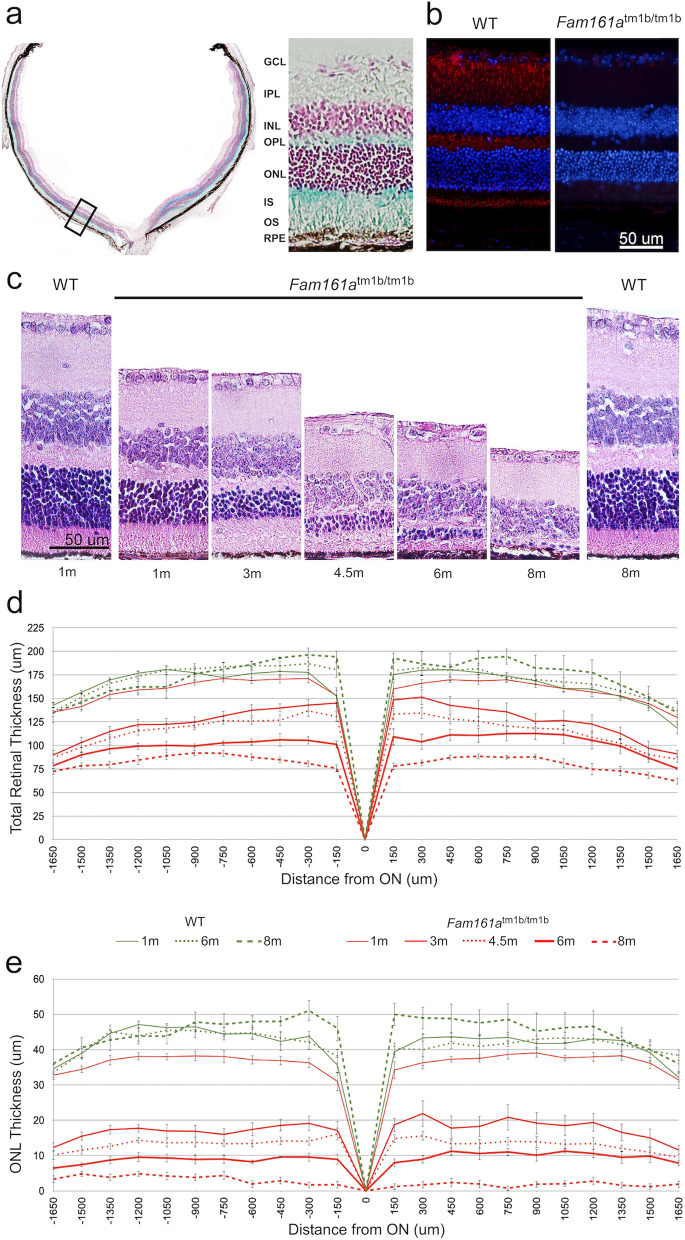
Retinal structure in *Fam161a*^tm1b/tm1b^ and WT mice. (**a**) LacZ expression in *Fam161a*^tm1b/tm1b^ mouse retina. LacZ cassette was inserted instead of exon 3 of *Fam161a*, and therefore the cells that express LacZ are the cells that originally expressed *Fam161a*. Blue color represents the area where LacZ was able to dismantle β-gal. Nuclei of different cells are stained in red. A black square (left) represents the region chosen for magnification (right side). (**b**) Representative IHC images from 1 month old *WT and Fam161a*^tm1b/tm1b^ mouse retinas stained with anti-Fam161a antibody. The retina was counterstained with DAPI to define nuclear layers. Original magnification: × 20. Scale bar: 50 μm. (**c**) Retinal histology in *Fam161a*^tm1b/tm1b^ and WT mice. Representative images of mid-peripheral retina stained with hematoxylin and eosin. Original magnification: × 40. Scale bar: 50 μm. (**d**) Total retinal thickness in *Fam161a*^tm1b/tm1b^ and WT mice. (**e**) ONL thickness in *Fam161a*^tm1b/tm1b^ and WT mice. The measurements in **d** and **e** panels were done in fragments of 150 µm from the optic nerve (ON) towards the nasal (N) and temporal (T) periphery.

To determine the expression pattern of *Fam161a* in the mouse retina without using antibodies, we utilized the LacZ cassette that was inserted into the mouse genome instead of exon 3. LacZ expression was identified mainly in the inner segment but also in the outer segment of photoreceptors and in the outer plexiform layer (Fig. 2a). We observed immunofluorescence staining that is similar but not identical to the one observed by using Fam161a antibodies. The staining we obtained was absent in the *Fam161a* KO retina (Fig. 2b). The LacZ results confirmed the previous^6,7^ immunohistochemistry staining findings (Fig. 2b) regarding the localization of the Fam161a protein in the mouse retina.

Retinal images of 83 *Fam161a*^tm1b/tm1b^ and 40 WT mice were stained with hematoxylin and eosin and analyzed using the Image-J software (Fig. 2c–e). H&E staining of retinal sections (Fig. 2c) demonstrated the gradual thinning of the ONL in *Fam161a*^tm1b/tm1b^ retina. Retinal thinning is statistically significant between 1 month-old mutant mice comparing to all other ages, while no statistically significant difference was obtained between1 month-old WT and mutant mice. When the ONL was studied, a statistically significant difference was obtained among all age groups. Progressive injury of the outer retina and thinning of ONL is observed from 3 months of age, and only sparse photoreceptor nuclei remain at the age of 8 months.

Progressive thinning of the whole retina and ONL occurred in *Fam161a*^tm1b/tm1b^ mice while WT showed stable retinal and ONL thickness over time. We observed thinning of the retina in the *Fam161a*^tm1b/tm1b^ mice groups compared to WT (Fig. 2d and Table 1): while mean of total retinal thickness was similar at age of 1 and 6 months in WT mice, a statistically significant decrease was obtained in the *Fam161a*^tm1b/tm1b^ mice along age reaching about 50% at the age 8 months (81.15 µm compared to 155.83 at the age of 1 month). Furthermore, we identified major and statistically significant thinning of the ONL, which explains the thinning of the retina (Fig. 2e and Table 1).

**Table 1:** Total retinal and outer nuclear layer (ONL) thickness in WT and *Fam161a*^tm1b/tm1b^ mice.

Genotype	WT		*Fam161a*^tm1b/tm1b^			
Age	1 month	6 months	1 month	3 months	6 months	8 months
Total retinal thickness (mean, µm) [p-value]	165.49	168.88	155.83 [0.016]	120.19 [< 0.001]	102.01 [< 0.001]	81.15 [< 0.001]
ONL thickness (mean, µm) [p-value]	46.2	41.1	35.4 [0.0002]	17.03 [< 0.001]	9.0 [< 0.001]	2.55 [< 0.001]

### Visual function of ***Fam161a***^tm1b/tm1b^ mice

**Figure 3:**
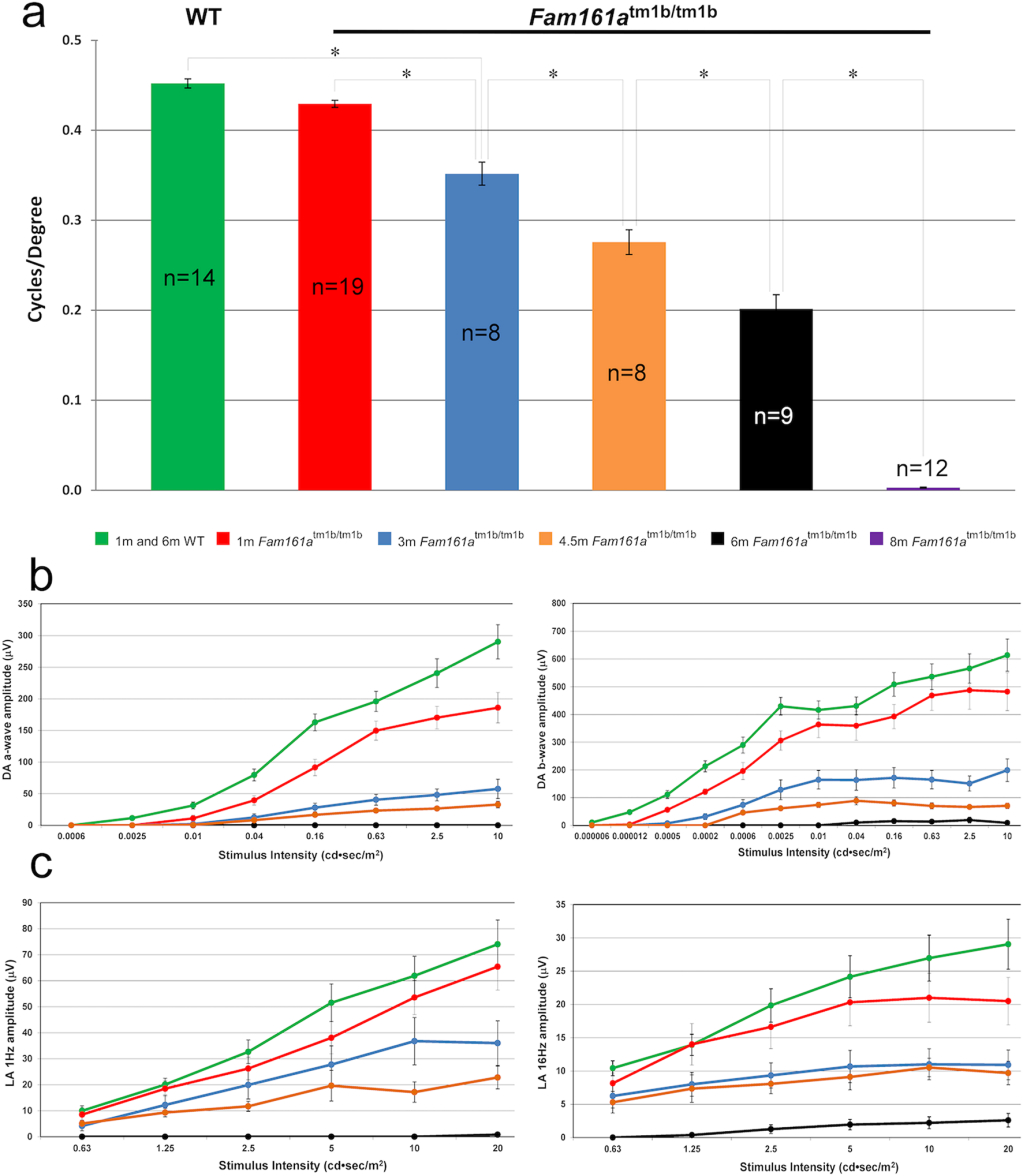
Retinal function in *Fam161a*^tm1b/tm1b^. (**a**) Visual acuity in WT (average results of 7 mice at the age of 1 months and 7 mice at the age of 8 months) and mutant mice in different ages (represented by the different colors). (**b**) A-wave and B-wave ERG responses in dark adapted conditions of WT and *Fam161a*^tm1b/tm1b^ mice at different ages. The green line represents WT mice at different ages (average results of 10 mice at the age of 1 months and 9 mice at the age of 8 months). Red, blue, orange and black lines represent mutant mice at ages 1, 3, 4.5 and 6 months respectively. (**c**) ERG responses in light-adapted conditions (1 Hz and 16 Hz stimulus) of WT and *Fam161a*^tm1b/tm1b^ mice at different ages.

Visual acuity (VA) in *Fam161a*^tm1b/tm1b^ mice was estimated by OKT test. VA deteriorates over time, while in WT mice VA remains stable through the 8 months of the experiment (Fig. 3a). At the age of 1 month, a small but not statistically significant difference between *Fam161a*^tm1b/tm1b^ and WT mice was observed, while a significant difference was obtained from the age of 3 months and on (p-value < 0.001, marked in *). At the age of 8 months *Fam161a*^tm1b/tm1b^ mice are not able to distinguish between light and dark.

Retinal function was assessed in parallel by electroretinography. Scotopic and photopic ERG responses deteriorate over time in *Fam161a*^tm1b/tm1b^ mice, while in WT mice the responses remain stable through 6 months of the experiment (Fig. 3b–c). At 1 month of age, the difference between WT (green line) and *Fam161a*^tm1b/tm1b^ (red line) is already notable. At the age of 6 months, (black line) *Fam161a*^tm1b/tm1b^ mice photoreceptor response to light stimulus is not detected by ERG**.**

### Retinal imaging of ***Fam161a***^tm1b/tm1b^ mice

**Figure 4:**
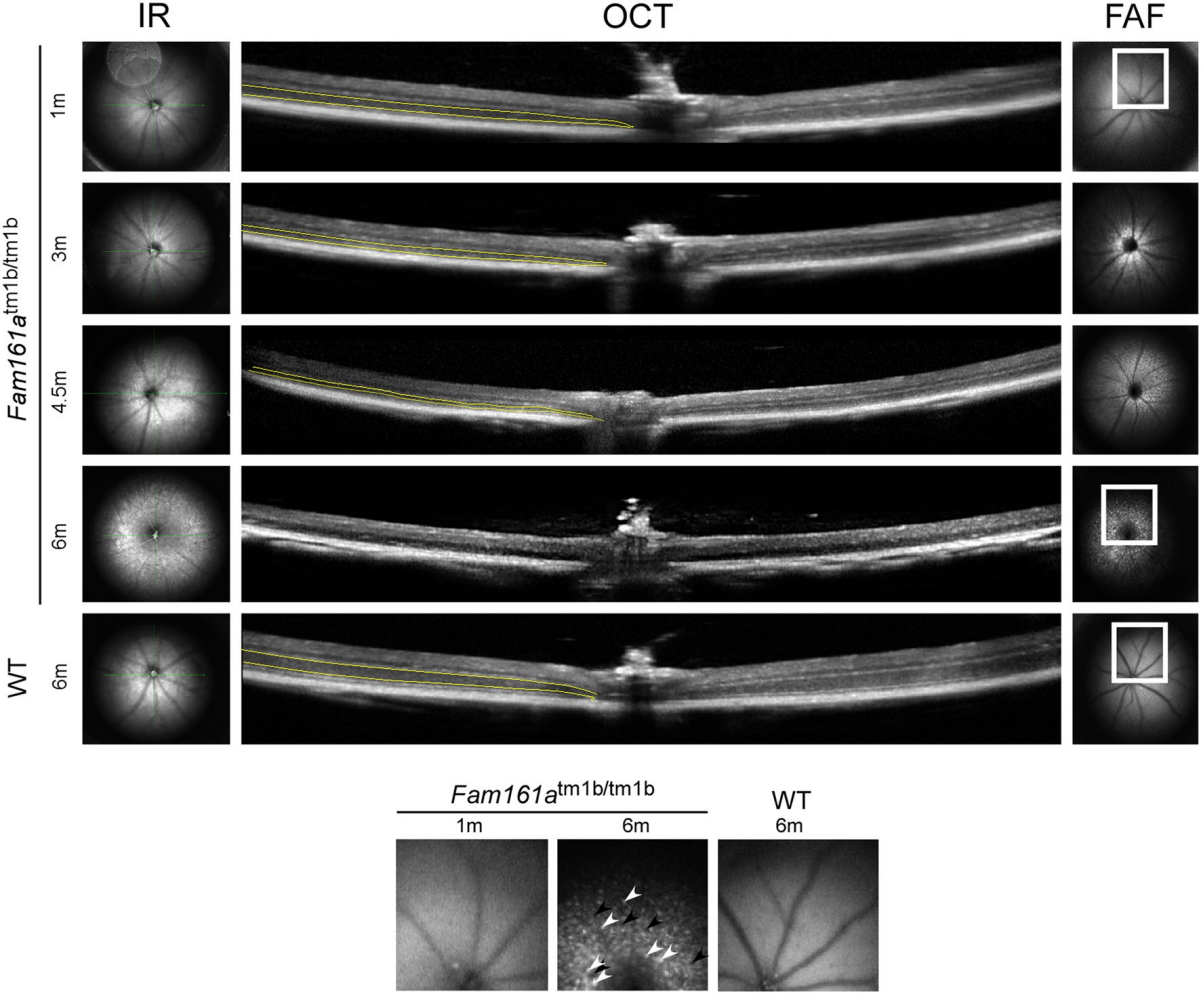
FAF and OCT imaging of *Fam161a*^tm1b/tm1b^ mouse retinas at different ages. A comparison between *Fam161a*^tm1b/tm1b^ mouse retinas at 1, 3, 4.5 and 6 months of age and WT mouse retina at the age of 6 months is presented. The outer nuclear layer which represents the photoreceptors is marked by yellow lines. Enlarged areas are highlighted in squares and are shown at the bottom of the figure. Patchy hypoautofluorescent spots are marked with black arrowheads and hyperautofluorescent spots are marked with white arrowheads.

In vivo imaging of *Fam161a*^tm1b/tm1b^ mice by fundus auto-fluorescence (FAF) revealed narrowing of blood vessels and formation of patchy hypo (black)-as well as hyperautofluorescent (white) spots that developed over time in *Fam161a*^tm1b/tm1b^ mice, indicating widespread retinal degeneration. These changes are most clearly seen at the latest time point (Fig. 4, bottom panel). OCT imaging showed thinning of the outer nuclear layer (ONL) in affected animals over time (marked by yellow lines in Fig. 4). At the age of 6 months the ONL was not detectable at all. WT mouse retina remained at the same thickness even at the age of 6 months (Fig. 4-bottom row).

### ***Fam161a***^tm1b/tm1b^ photoreceptor structure

**Figure 5:**
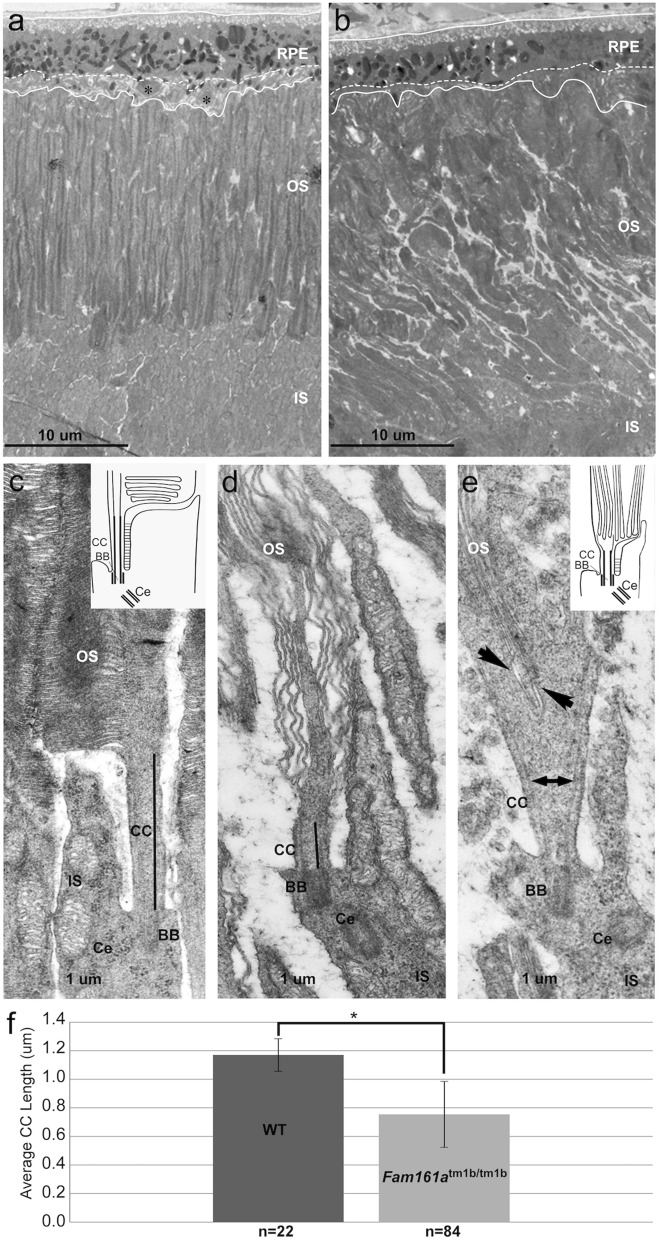
TEM (Transmission electron microscopy) sections of WT and *Fam161a*^tm1b/tm1b^ retina. (**a**) Representative retina of WT at 1 month. (**b**) Representative retina of *Fam161a*^tm1b/tm1b^ at 1 month. The two solid lines indicate the extension of the RPE cells. The dashed lines indicate the apical surface of the epithelium from which the microvilli-like extensions extend towards the photoreceptor outer segments. Black asterisk indicate engulfed disk stacks released from outer segments. RPE-Retinal pigment epithelium, OS-Outer segment, IS-Inner segment. Scale bars: 10 µm. (**c,d**) Photoreceptor morphology of 1 month-old WT (**c**) and *Fam161a*^tm1b/tm1b^ (**d**) mice. A schematic drawing of the cell is presented on the top right on panel c. CC-connecting cilium (marked in black line), BB-basal body, Ce-adjacent centriole. Scale bars: 1 µm. Note that in the *Fam161a*^tm1b/tm1b^ photoreceptor outer segment disks are disorganized in a perpendicular orientation and the CC is shortened (marked in black line). (**e**) Photoreceptor morphology of a 1-month-old *Fam161a*^tm1b/tm1b^ mouse. Outer segment base is widened and microtubules spread in their upper part (double headed arrow). Outer segment disks are growing from the middle of the CC (arrowheads). A schematic drawing of the cell is presented on the top right. Scale bars: 1 µm. (**f**) Connecting cilium length analysis. Comparative analysis of WT CC length and *Fam161a*^tm1b/tm1b^ CC length (*P < 0.005, error bars represent SD).

TEM analysis of 1-month old WT and *Fam161a*^tm1b/tm1b^ mouse retinas were performed in order to better understand the course of the disease at the cell level. In WT animal, all retinal layers and cells are well organized, densely packed, and have clear directionality. The outer segments are arranged upright, tightly packed and clearly distinguishable from the inner segment (Fig. 5a). In *Fam161a*^tm1b/tm1b^ mouse retina, disorganization is evident along the section and it is difficult to distinguish between different cell regions because cells invade to neighbor ones (Fig. 5b). The outer segments are disorganized, lost their polarity, and spaces are formed between them.

Higher resolution analysis of a single rod photoreceptor from WT retina (Fig. 5c) shows a typical feather shape, the disks of the outer segment are well organized and grow horizontally and parallel to each other, the structures of the connecting cilium are also parallel to each other and on the base of the connecting cilium, basal body and the centriole are evident. Rod photoreceptors from the *Fam161a*^tm1b/tm1b^ mouse retina (Fig. 5d–e), however, show gross abnormalities in cell structure. Some photoreceptors had a very short connecting cilium, the disks of the outer segment are growing vertically out of it, and the inner segment also lost its structure (Fig. 5d). Other photoreceptors show dysmorphic connecting cilium which becomes wider at the end that is closer to the outer segment (Fig. 5e). The disks start to grow from the middle of the connecting cilium and the structures of the connecting cilium are not parallel any more. The inner segment also lost its structure. In all cases photoreceptors lost their feather shape and shortening of the connecting cilium and dysmorphic inner segment and outer segment are evident. The connecting cilium length is reduced in *Fam161a*^tm1b/tm1b^ photoreceptors (WT: 1.170 ± 0.11 µm; *Fam161a*^tm1b/tm1b^: 0.754 ± 0.23 µm; Fig. 5f).

**Figure 6:**
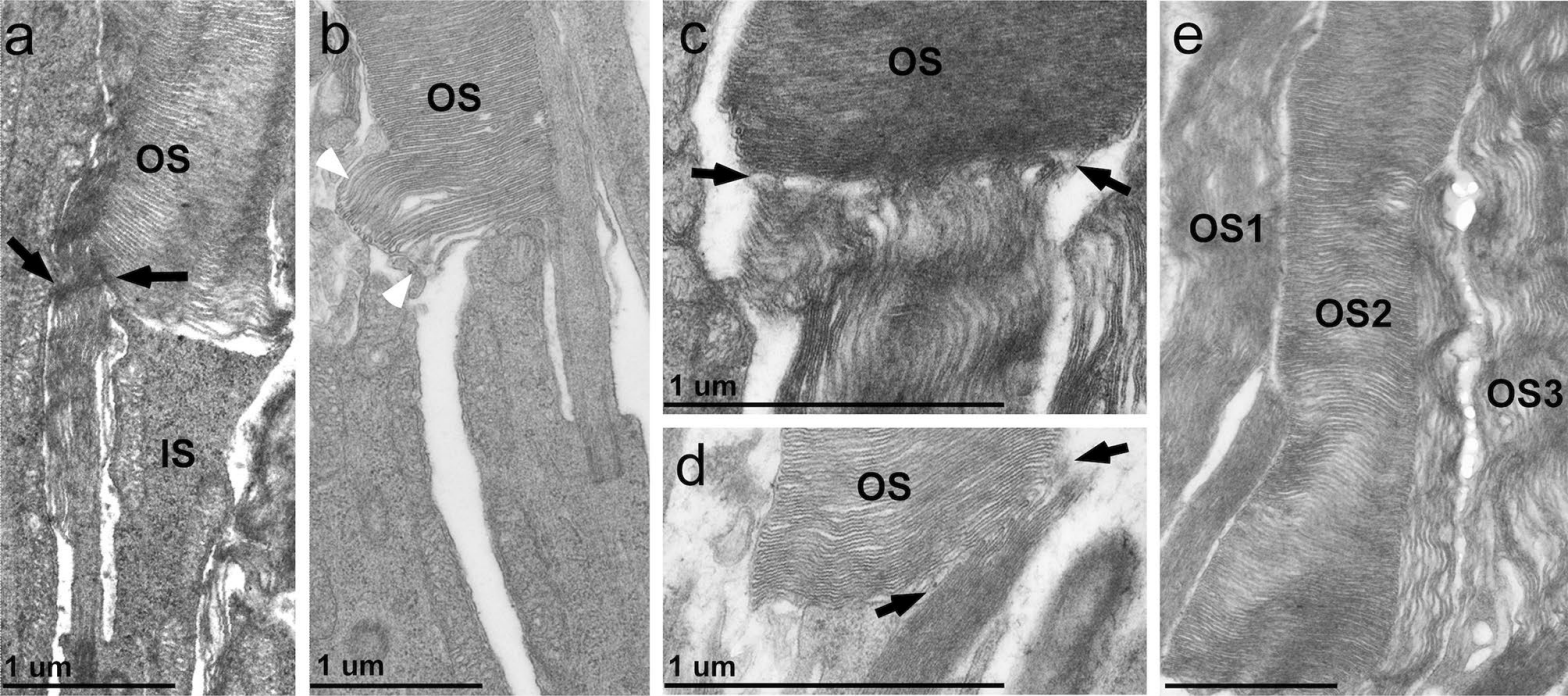
High-resolution TEM analysis of *Fam161a*^tm1b/tm1b^ retina. a single photoreceptor cell from 1-month-old *Fam161a*^tm1b/tm1b^ mouse representing different processes. (**a**) New discs with different orientation are marked in black arrows. Scale bar: 1 um. (**b**) Overgrow of disks is marked by white arrowheads. Scale bar: 1 um. (**c**,**d**) Zoom-in into the exact location where the orientation of the discs changes (marked in black arrows). (**e**) Different morphology in the same retina at the age of 1 month is observed. Three different outer segments are shown (OS1, OS2, OS3), and one of them (OS2) shows correct morphology.

At the age of 1 month, most photoreceptors in the *Fam161a*^tm1b/tm1b^ retina were dysmorphic, but in some photoreceptors we were able to observe the process of degeneration (Fig. 6a–d). We identified *Fam161a*^tm1b/tm1b^ dysmorphic outer segment disks that change orientation, starting from the position that is closer to the inner segment, where they are growing from, whereas in the position that is closer to the RPE (where they are aging and disconnecting from the photoreceptor) the outer segment disks are still in the normal shape (Fig. 6a-b).

Additional interesting observation was seen at the macro level in all mice that were examined at the age of 1 month. In different areas of the retina, photoreceptors at different stages of degeneration were observed, located near each other (Fig. 6e). Minority of the photoreceptors had still correct morphology, while their neighbors showed disrupted and dysmorphic outer segment.

### Cellular and molecular degenerative markers in ***Fam161a***^tm1b/tm1b^ retina

Microglia have been previously shown to be activated during retinal degeneration in different retinal degenerative processes, including in the *Fam161a* gene trap model^20^. In the 1-month-old WT retina, microglia positive for the IBA1A antigen are situated in the INL and present discrete and thin processes (Supplementary Fig. S1). No cells are detected in the ONL at this age, nor later (data not shown). In contrast, in the 1-month old *Fam161a*^tm1b/tm1b^ mouse retina, IBA1A-positive microglia are already detected in the ONL with processes elongating from the INL and invading a large distance in the ONL. At 2 months, the processes are thicker and at 3 months, positive cells accumulated in the ONL with a denser morphology and shorter processes (Supplementary Fig. S1).

**Figure 7:**
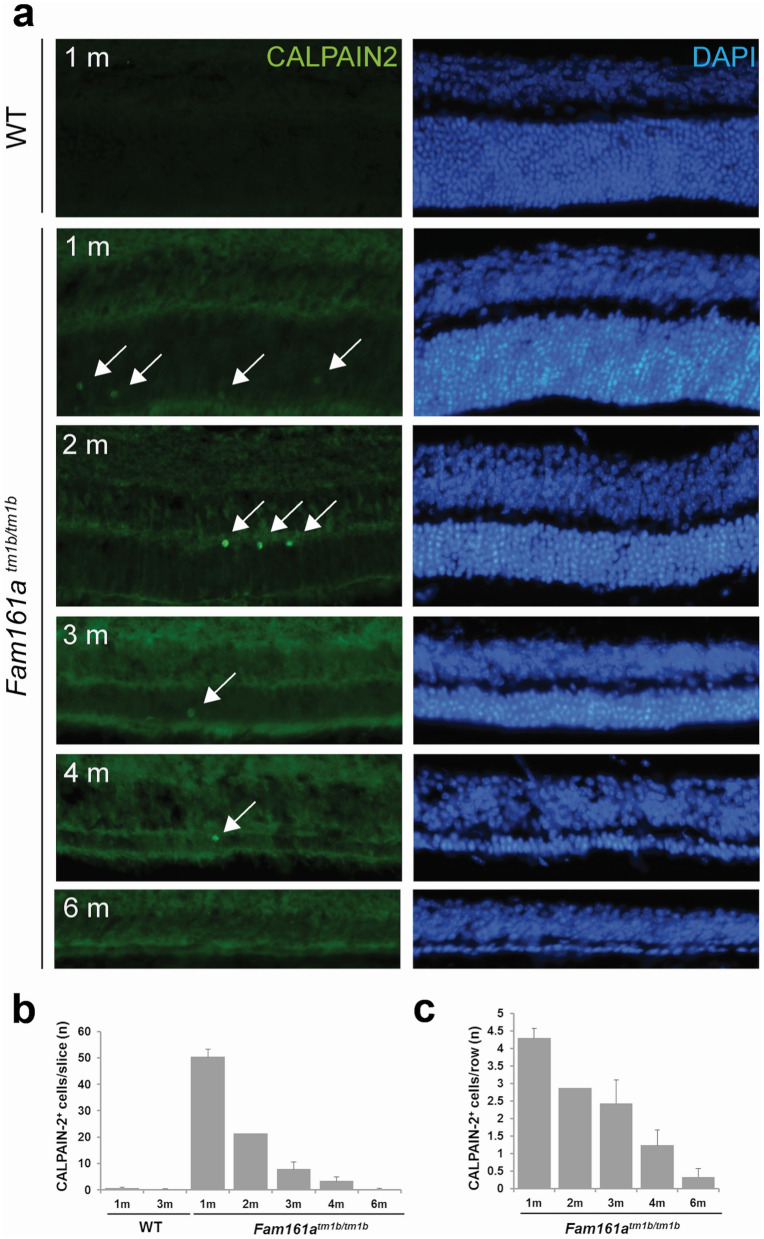
CALPAIN-2 is more present during the early stages of retinal degeneration. (**a**) CALPAIN-2 (in green, arrows) is present in the ONL of *Fam161a* ^tm1b/tm1b^ mouse all along the degenerative process, but the enzyme is almost absent in the WT retina (bottom). (**b**) The quantification of the absolute number of CALPAIN-2-positive cells shows that cell death is more prominent at 1 month. (**c**) The rate of the degeneration, evidenced by the number of CALPAIN-2-positive cells per row of photoreceptor, linearly declined with time. 1 m: 1 month. n = 3 for all time points, with the exception of 2 month old mice (n = 2).

Rod degeneration in inherited retinal degeneration is often non-apoptotic and we investigated whether CALPAIN-2, which was recently shown to tightly correlate with rod death events^34^, also highlights the degenerative process of a retinal ciliopathy such as the *Fam161a*^tm1b/tm1b^ mouse. Very rare CALPAIN-2 positive cells were observed in the ONL of WT retina, whereas CALPAIN-2 is markedly present in many cells at the age of 1 month in the KO mouse retina and the number of CALPAIN-2-positive cells decreased with time (Fig. 7a,b). When this number is expressed as a function of the number of row nuclei, a linear decrease of the cell death rate is apparent with the increasing age of the *Fam161a*^tm1b/tm1b^ mouse (Fig. 7c).

## Discussion

In this study we characterized retinal degeneration progress in a new *Fam161a* KO (*Fam161a*^tm1b/tm1b^) mouse model lacking exon 3. Our functional and structural findings indicate that there is a progressive retinal degeneration that starts before the age of 1 month, similar to the previous mouse model (*Fam161a*^*GT/GT*^)^20^. In our model, complete degeneration of photoreceptors cell layer is evident approximately at the age of 8 months, when no ERG or behavioral responses to light stimulus are detected. The degeneration in our mouse model is slightly slower than the previously reported model of *Fam161a*^20^ but faster than many other reported models such as rd6, rd7 rd8 and rd9^14^. In the previously reported GT model, at the age of 3 months, mice had about 3 rows of photoreceptor nuclei and 1 row at the age of 6 months, while in *Fam161a*^tm1b/tm1b^ mouse model we present here, these stages appear about 1–2 months later. The reason for this difference in severity is currently unclear. It might stem from background genetic differences between mouse strains or the truncated form of Fam161a which is expressed in *Fam161a*^GT/GT^ mice^20^ might have a negative effect on photoreceptor survival.

The results of the RT-PCR analysis demonstrate that only a short mRNA transcript is produced, including only exons 1, 1a, and 2 while exons 3 (the largest exon that includes most of the disease-causing mutations in humans) and downstream exons are missing. The model we describe here is different from the previously reported GT model, where RNA is produced approximately at the same level as in WT mice^20^. In addition, in the previous model, low amount of truncated protein was identified by immunohistochemistry staining while no protein at all was identified by Western blot analysis, which led to the conclusion that this model may have residual function^20^. In the current model, only a short RNA molecule including exons 1–2 has been identified which does not contain the most important functional domains of the protein^10,13^ and therefore no functional protein is likely to be produced. In addition, immunohsitochemical staining with antibodies against the protein region encoded by exon 3 and 5 (without exon 4) of *Fam161a* showed no expression in *Fam161a*^tm1b/tm1b^ mice, indicating that there is most probably no functional protein. Since the transcript/s produced in *Fam161a*^tm1b/tm1b^ mice do not include the main exon (#3) as well as the end of the open reading frame, we believe that this *Fam161a*^tm1b/tm1b^ mouse is not leaky, and therefore no full-length protein is produced.

LacZ staining demonstrated that the construct that was inserted is likely to be under the control of the *Fam161a* promoter and expressed in similar but not identical locations where *Fam161a* has been reported to be expressed, supporting previous immunohistochemical staining results^6,7,9,20^. While previous and our immunohistochemical staining identified the protein mainly in the photoreceptors, but also in the OPL and less in the IPL, LacZ staining showed expression only in photoreceptors and OPL.

OKT responses were dramatically reduced by more than 50% in *Fam161a*^tm1b/tm1b^ mice comparing to WT group at the age of 6 months and therefore are correlated with the full-field ERG analysis which revealed progressive deteriorations along time both in scotopic and photopic conditions. The model we present here demonstrates slower reduction of the ERG responses in comparison to *Fam161a*^GT/GT^ mouse model at the initial disease phase, which exhibits abnormal scotopic ERG responses already at 1 month of age, and almost undetectable responses at 4 months^20^.

Our in vivo imaging analysis, which was performed in *Fam161a*^tm1b/tm1b^ mouse model for the first time, demonstrated an increased number of hyper and hypo auto-fluorescent spots. We assume that the spots might represent accumulation of microglial cells^35^. These spots provide a valuable prediction of wide-spread retinal degeneration as we also see in other mouse model for retinal degeneration^14^ and human patients deficient for FAM161A who suffer from retinal degenerative diseases in the clinic^6^.

In addition, retinal morphometry and measurements revealed that the ONL thickness was reduced from the age of 1 month old to the age of 8 months old by more than 90% (from 35.4 to 2.55 µm) and total retinal thickness was reduced from the age of 1 month old to the age of 8 months by nearly 50% (from 155.83 to 81.15 µm) In *Fam161a*^GT/GT^ mouse model only the reduction of the ONL was measured and it was faster than in *Fam161a*^tm1b/tm1b^ model and compatible with the thinning of the retinal layers as they were observed in H&E staining^20^. Based on our histological analysis, we predict that the most dominant and aggressive loss of photoreceptor cells in the ONL occurred between the ages of 1–4.5 months in *Fam161a*^*t*m1b/tm1b^ mice. Regarding the oldest group, only sparse photoreceptor nuclei remained at the age of 8 months, which probably are barely detectable by functional tests. We assume that most of the nuclei we observe at the age of 8 months belong to cells that are not functional anymore and hence we are not able to detect any responses in the VA or ERG tests. In other ages, we observed that the functional tests (VA and ERG) are in-line with the structural ones (OCT, FAF and histological measurements).

The results of the TEM analysis that we performed support those reported in the *Fam161a*^GT/GT^ mouse model regarding the shape of the cilia and the photoreceptors. In both models photoreceptor outer segment disks were disorganized in a perpendicular orientation. The outer segment base was wider and shorter than in WT mice. This indicates that Fam161a participates in disk morphogenesis at the base of the outer segment as found in other RP models with defective disk formation^36^. In addition, we observed different morphology of photoreceptors in the same mouse at the age of 1 month, while several photoreceptors showed disrupted and dysmorphic discs, other photoreceptors in the same observed area seems to have normal morphologic structure at the observed time point. Those results indicate that near cells that undergo degeneration (and may not be cured even at this early timepoint), one can find cells with normal structure which did not begin the degeneration process yet. Those remaining functional cells probably can be rescued by gene therapy or other intervention.

In addition, we observed diverse arrangements of the membrane disk stacks in one and the same outer segment (e.g. Fig. 6) which is in line with findings in other rd models^37^. Almost nothing is known about the physiological or molecular basis of this phenomenon. One reason may be that the renewal of the outer segment disks does not occur in a continuous temporal sequence. At the base of the outer segment, new membrane disks are formed, which are mainly stacked in a period of 1–2 h after the light is switched on^38^ and at that time disk neogenesis is likely faster than at other times. However, there are also indications that not only the speed of disk morphogenesis, but also the molecular composition of the disks along the outer segment may periodically change. More recent, still controversial discussed data indicates that rhodopsin and peripherin-2 are incorporated into the disk stacks under different physiologic conditions, preferentially in dark or light, respectively^36,39^. Although we currently do not know at which period Fam161a participates at the of disk neogenesis it is conceivable that Fam161a´s absence may differential affect disk stacking under different physiologic conditions in a periodic manner.

The heterogeneity of photoreceptor alteration was also observed at the molecular level, with sporadic appearance of the CALPAIN-2 protein in photoreceptors. CALPAINS were shown to be a good indicator of cell death in different forms of inherited retinal degenerations^34,40^ . Because certain CALPAIN inhibitors have shown to delay photoreceptor degeneration in *Rd1* retinal explants^41^, the rare observation of CALPAIN-2 positive cells in the *Fam161a*^tm1b^^/tm1b^ model and the linear decrease of cell death rate with aging also suggest that many photoreceptors can be rescued by gene therapy or a neuroprotective approach. However, the marked invasion of microglia in the ONL at the age of 3 months rather suggests an accentuated deterioration of the photoreceptors, which may render more difficult therapeutic applications.

Our results indicate a functional role of *Fam161a* in the retina, specifically in the photoreceptors. Conclusively, the decrease in all parameters that was noted in *Fam161a*^tm1b/tm1b^ mice during the period of the experiment, showed both that the functional and structural degenerations are less aggressive than those observed in some other genetically-determined retinal degeneration mouse models such as rd1, rd4 and rd10 mice^14,42,43^ and may better resemble the course of RP in humans. Currently, gene augmentation therapy is being examined in this model, as a possible first step to future application of such treatment in human patients with RP caused by *FAM161A* biallelic mutations.

## Methods

Experiments were conducted in compliance with the ARVO Statement for the Use of Animals in Ophthalmic and Vision Research and were approved by the Hebrew University animal ethics committee.

### Generation of Fam161a KO mouse

Mice were generated by Knockout Mouse Project (KOMP) Repository. Promoter-driven selection cassette that contains FRT and LoxP sites and a reporter gene *LacZ* (Fig. 1a) was introduced to C57BL/6 N embryonic stem cells, which were later injected into b6(Cg)-Tyr^c-2 J^/J blastocysts. The resulting chimeras were bred with C57BL/6 J mice in order to receive heterozygous mice for the non-activated construct (Fam161a^tm1a(KOMP)Wtsi^). These mice were shipped to our animal facility for further breeding and analyses. Activation of the cassette was performed by crossbreeding heterozygous mice with B6.C-Tg(Pgk1-cre)1Lni/CrsJ mice (JAX stock #020811) to receive mice with *LacZ* instead of exon 3 of Fam161a (Fam161a^tm1b(KOMP)Wtsi^). This activation results in a short RNA transcript which does not contain the major functional protein domain and is likely to be true null. Heterozygous *Fam161a*^tm1b/WT^ mice were generated on a C57BL/6 N background and intercrossed to establish homozygous male and female cohorts for initial phenotypic screening. *Fam161a*^tm1b/WT^ founders were screened for known mutations in *Crb1* (rd8)^44^, *Rpe65* (rd12)^45^, *Gnat2*^46^ and *Pde6b* (rd10)^42^ (rd1)^43^ (Primers sequences in Supplementary Table S1). The rd1 and the rd8 mutations, that is common in 6 N strain^43,47^, were identified in these mice, and they were crossed with wild-type C57BL/6 J to establish mutant mice free of the confounding rd1 and rd8 mutations. Ten generations of *Fam161a*^tm1b/WT^ were crossed with C57BL/6 J mice to establish a strain that has clear and identical background. A research colony was bread crossing heterozygotes to produce homozygous mutants and wild-type control littermates for this study.

### Animals

Animals were maintained at the animal facility of the Hebrew University-Hadassah medical center, Ein Kerem campus, Jerusalem, Israel, and kept under specific pathogen free and 12-h light/dark conditions. General microbial examinations were performed routinely by the animal research facility staff. The weight, development, growth and behavior of the knockout animals was also routinely checked and appeared to be normal.

Retinal development and progression of retinal degeneration was studied at the ages of 1, 3, 4.5, 6 and 8 months. Mice were anesthetized by intraperitoneal injections of a mixture of 0.85 µl Ketamine (Bedford Laboratories, Bedford, OH) and 0.15 µl Xylazine (VMD, Arendonk, Belgium), diluted with saline 1:10 and injected with doses suitable to their body weight (1 µl/g). Pupils were dilated with 1% tropicamide and 2.5% phenylephrine, local anesthetic drops were administered (benoxinate HCl, 0.4%; all ocular drops from Fisher Pharmaceuticals, Tel-Aviv, Israel), and eyes were lubricated with methylcellulose prior to electroretinography (ERG), fundus photography and optical coherence tomography (OCT). Before sacrifice, animals were anesthetized by over-dose of Ketamine and Xylazine followed by cervical dislocation.

### Genotyping

DNA was extracted from mouse ear punches using 180 µl of 50 mM NaOH to 5–10 mg of mouse ear sample and incubated for 10 min at 95 °C. Extract was neutralized by adding 20 µl of 1 M Tris–HCL (pH 8.0). Genotyping was performed by using KAPA Mouse Genotyping Kit (KAPA Biosystems), genotyping primers targeted the WT allele, *LacZ* insert, exon 3 region are reported in Supplementary Table S1.

### RNA isolation and RT-PCR analysis

RNA was extracted from mouse retinas using TRI Reagent (T9424, Sigma-Aldrich, Rehovot, Israel). RNA was converted to cDNA using Verso cDNA Kit (AB-1453/B, Thermo Scientific). RT-PCR was performed with 50 ng cDNA to amplify intron-spanning fragments between all *Fam161a* exons. Primers of the reactions are shown in Supplementary Table S1.

### Optokinetic tracking (OKT) response

Visual acuity was measured using an optokinetic testing apparatus (OptoMotry; Cerebral Mechanics, Inc., Lethbridge, AB, Canada) by recording the tracking response (optokinetic reflex) to a rotating visual stimulus displayed on four LCD panels surrounding the mouse. Visual acuity was measured at 100% contrast in n = 8–19 mice per age group.

### Electroretinography

Full field ERG was performed in anesthetized animals (n = 8–19 per age group) after overnight dark adaptation using a Ganzfeld dome and a computerized system (Espion E2, Diagnosys LLC, Littleton, MA), as previously described^48^. Briefly, pupils were dilated and gold-wire active electrodes were placed on the central cornea. A reference electrode was placed on the tongue and a needle ground electrode was placed intramuscularly in the hip area. Dark-adapted rod and mixed cone-rod as well as light-adapted 16 Hz cone flicker responses to a series of white flashes of increasing intensities (0.00008–9.6 cd s/m^2^) were recorded. All ERG responses were filtered at 0.3–500 Hz, and signal averaging was applied.

### Optical coherence tomography, fundus auto-fluorescence and fundus photography

Retinal structure was studied in vivo by optical coherence tomography (OCT), fundus auto-fluorescence (FAF) imaging and fundus photographs were taken (SPECTRALIS, Heidelberg). The procedures were performed in anesthetized animals with dilated pupils and lubricated eyes as mentioned earlier. At least 8 eyes from 8 different mice were examined per age group.

### Histology and immunohistochemistry (IHC)

Eyes were enucleated, fixated in Davidson solution for 8 h at 4 °C, and moved to 70% ethanol over-night at 4 °C. Eyes were incubated in a 80–> 90–> 100–> 100% ethanol gradient in room temperature for 30 min per each concentration, placed for 15 min in ethanol 100%:Xylene (1:1) solution in room temperature and washed twice with xylene 100% for 20 min each wash. Samples were incubated in paraffin (Paraplast Plus, Leica biosystems) at 58 °C for 3 times, each incubation lasted 40 min, later they were embedded in paraplast, and serially cut at 5 μm thickness sections through the center of the optic nerve. For immunohistochemistry, standard immunohistological analysis protocol for paraffin section was carried out, as previously described^49^, commercial buffer (ImmunoRetriever 20 × with citrate pH 6.62, Bio Sb) and commercial antibody (rabbit anti-Fam161a, 1:500, HPA032119, Sigma-Aldrich) were used, recognizing an epitope in a sequence encoded by exon 3 and 5 corresponding to short isoform in which exon 4 is skipped). For descriptive histology and quantitative analysis, sections were stained with hematoxylin and eosin (H&E) following a standard protocol. Each retina was divided to sections of 150 μm from the optic nerve towards the nasal and temporal periphery, and thickness of each retinal layer was measured separately once. Then results from the corresponding retinal areas were averaged and a statistical analysis was performed using t-test (significance of p < 0.05).

For immunohistochemistry, commercial antibodies were used (anti-IBA1, 1:600, #019–19,741, WAKO and anti-CALPAIN-2, 1:200, ab39165, Abcam) to characterize the process of cell death at different ages.

### X-Gal staining

Eyes were enucleated from *Fam161a*^tm1b/tm1b^ and WT mice at the age of 1 month and then fixed in 4% paraformaldehyde for 20 min at room temperature. Cornea and lens were removed, eyecup were cryoprotected in a 10–> 20–> 30% sucrose gradient at 4 °C for at least 1 h per each concentration. Eyecup were embedded in optimum cutting temperature medium (Sakura Finetek USA, Torrance, CA, USA) frozen in dry ice and 2-methylbutane slurry, and sectioned at 10 µm onto Superfrost Plus (Thermo Fisher) slides with a cryostat (Leica CM1950, Leica, Wetzlar, Hesse, Germany). Slides were washed with PBS and stained for X-gal with β-Galactosidase Reporter Gene Staining Kit (Sigma-Aldrich, Rehovot, Israel) followed by PBS washing and nuclear fast red staining (Vector Laboratories, Burlingame, CA, USA) according to the protocols supplied by the companies. After an additional PBS wash, slides were coverslipped with Vectamount (Vector Laboratories, Burlingame, CA, USA) and imaged.

### Statistical analysis

For all histological quantifications, at least 8 *Fam161a*^tm1b/tm1b^ mice were imaged for each age and in addition 8 WT mice at the age of 1, 6 and 8 months. Measurements were combined to determine the average per each group. For CALPAIN-2-positive cell quantification, n = 3 per age and 3 retina slices at the proximity of the optic nerve were counted per animal. For visual acuity and histology, pairwise analyses were performed by using t-test, and p-value of 0.05 or less was considered as statistically significant results.

### Imaging

All observations and photography were performed using an Olympus BX41 microscope equipped with a DP70 digital camera. Image processing and quantification were performed using Adobe Photoshop CS2 and Image-J softwares.

### Fixation for conventional electron microscopy

Eyes were enucleated and fixed in 2.5% glutaraldehyde in 0.1 M cacodylate buffer (pH 7.4) and 0.1 M sucrose for 30 min in room temperature. Cornea and lens were removed and the eye cups were incubated in the same mixture for an additional 1.5 h. Eye cups were washed 5 times with 0.1 M cacodylate buffer containing 0.1 M sucrose for 30 min. Later, eye cups were fixed with 2% OsO_4_ in 0.1 M cacodylate buffer with 0.1 M sucrose for 1 h at room temperature. Samples were washed in water and dehydrated in ascending ethanol concentration (2 times per concentration, 10 min each) 30–> 50–> 70–> 80–> 96–> 100% ethanol. Samples were embedded in Renlam M-1 restin (Serva Electroporesis, Heidelberg, Germany)^20^.

Ultrathin sections were made by using Reichert Ultracut S ultramicrotome (Leica), collected on Formvar-coated copper grids and counterstained with 2% uranyl acetate in 50% ethanol and aqueous 2% lead citrate. The sections were analyzed in a Tecnai 12 BioTwin transmission electron microscope (FEI, Eindhoven, The Netherlands), as previously described^20^. Images were obtained with a CCD camera (charge-coupled-device camera, SIS MegaView3, Surface imaging system, Herzogenrath, Germany) and processed with Adobe Photoshop CS2 and Image-J softwares.

## Supplementary information

Supplementary Information.

## Data Availability

The datasets generated and/or analyzed in the current study are available from the corresponding author upon reasonable request.
