# Supplementary material for: A new mouse model for retinal degeneration due to Fam161a deficiency
Source: Sci Rep. 2021 Jan 21;11:2030. doi: 10.1038/s41598-021-81414-1 (PMC7820261; doi:10.1038/s41598-021-81414-1)
Supplement: Supplementary file 1 — Supplementary Information. [file 41598_2021_81414_MOESM1_ESM.docx]

**A new mouse model for retinal degeneration due to *Fam161a* deficiency**

Avigail Beryozkin^1^, Chen Matsevich^1^, Alexey Obolensky^1^, Corinne Kostic^2^, Yvan Arsenijevic^2^, Uwe Wolfrum^3^, Eyal Banin^1*^, Dror Sharon^1*^

^1^ Department of Ophthalmology, Hadassah Medical Center, Faculty of Medicine, The Hebrew University of Jerusalem, 91120 Jerusalem, Israel.

^2^ Department of Ophthalmology, Jules-Gonin Eye Hospital, University of Lausanne, 1004 Lausanne, Switzerland

^3^Institute for Molecular Physiology, Johannes Gutenberg University, 55128, Mainz, Germany.

**Supplementary Figure S1**

**
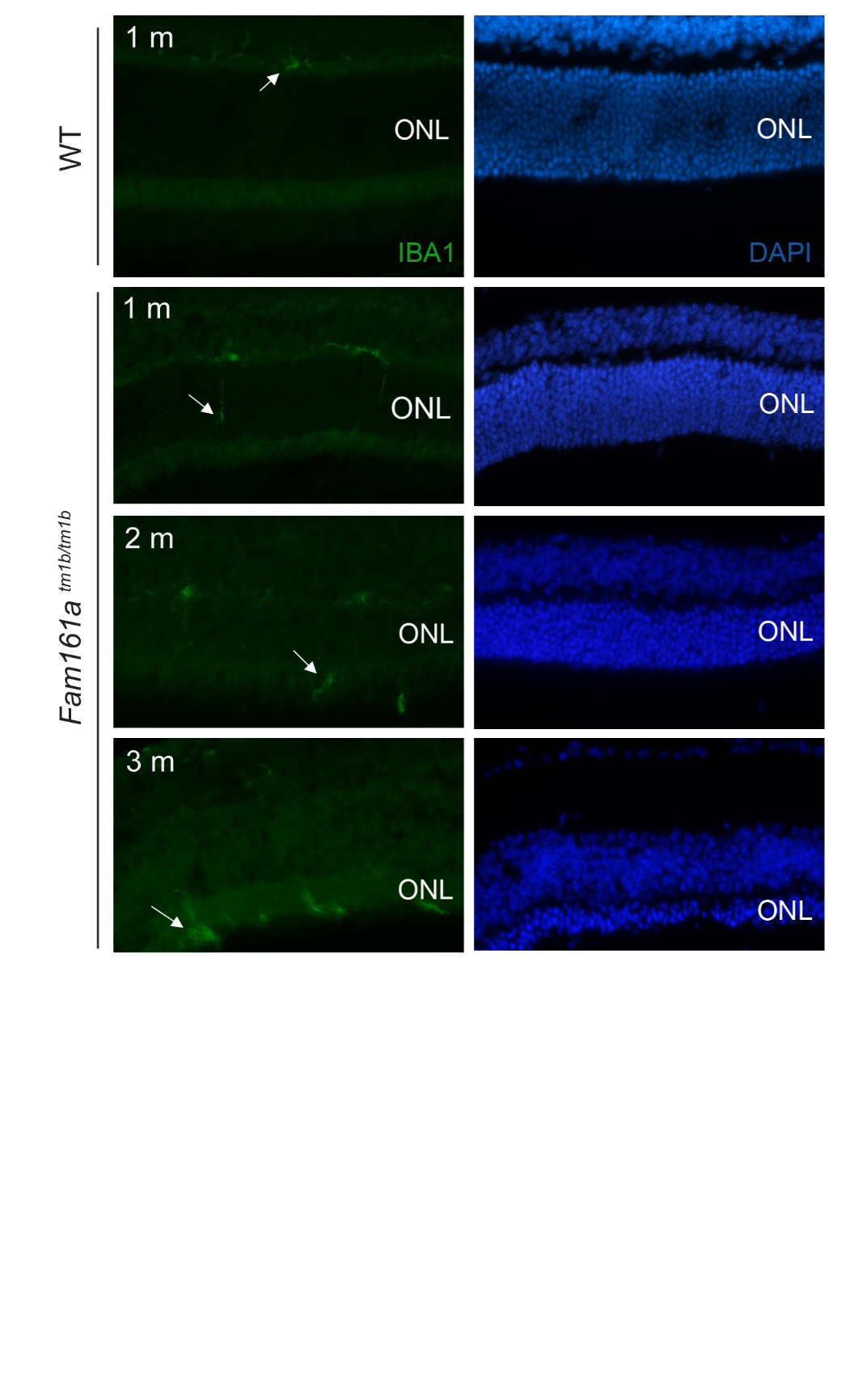
**

**Supplementary Figure S1:** Microglia invades progressively the photoreceptor layer during retinal degeneration of the *Fam16*1 ^tm1b/tm1b^ retina. Microglia activation is followed using the labelling against the IBA1 antigen. In wild type (WT) mouse retina, rare IBA1-positive cells (white arrow) are present between the interneurons and the outer nuclear layer (ONL). Such observation was made in 1- to 3-months-old retina. In 1-month-old retina of *Fam161a* ^tm1b/tm1b^ mice (KO), IBA1-positive cells present longer processes migrating into the ONL. At 2 months of age, some cells are present in the ONL and at 3 months more cells invade the photoreceptor layers. Note, that the morphology of the IBA1-positive cells is different with thicker processes.

**Supplementary Table S1:** Primer sequences used in this study

| Reaction name | | Forward primer | Revers primer | Length (bp) |
| --- | --- | --- | --- | --- |
| Mutations specific for 6N and 6J strains | CRB1 ex9 | gcacaatagagattggaggc | tgtctacatccacctcacag | 386 |
|  | RPE65 ex13 | catatgctagtcaagtaaagcag | cccaacccctttcctaatagag | 457 |
|  | GNAT2 ex5 | accgatgccaccttcttttt | tgctgtgagacctgagatgc | 265 |
|  | PDE6B ex13 | ggctctgatatggtgctgtg | aaagactcaccctaaggacgc | 453 |
|  | PDE6B ex16 | gacagagtaggccctgagagtc | cctgggattagggaggtctg | 655 |
|  | PDE6B  insertion | tgacaattactccttttccctcagtctg | gtaaacagcaagaggctttattgggaac  tacccacccttcctaatttttctcacgc | 398  587 |
| Genotyping of the strain | Wt | taaatgtgagaattcccttggcggc | gtcaccatcttgaccatgtctgacc | 576 |
|  | Ex 3 | gcccaggagctcataaacaa | ggactgcttgcttctgttcc | 421 |
|  | Post cre | gctaccattaccagttggtctggtgtc | tctgtgtacaagatcctggctttgg | 657 |
| RT-PCR for RNA sequences | 1F+1aR | cccagtacgaaagcgaagac | ccagcctggtttatggaatg | 295 |
|  | 1F+2R | cccagtacgaaagcgaagac | ctcgaatgccacctcttcat | 416 |
|  | 2F+3R | aaagacctgaaggctgtcca | ggtcactgacaaggcaggat | 171 |
|  | 3F+3R intra exonic | gcccaggagctcataaacaa | ggactgcttgcttctgttcc | 421 |
|  | 3F+4R | gtctcctaggcgcaagtcac | gggagccataagccttaacc | 364 |
|  | 4F+4R | tcccttgaggaaaagaaaatg | gggagccataagccttaacc | 168 |
|  | 4F+5R | tcccttgaggaaaagaaaatg | tcaaacaacatcggcctctt | 268 |
|  | 3F+5R | gtctcctaggcgcaagtcac | tcaaacaacatcggcctctt | 211  356 |

**Supplementary Note**

**Exon 1a sequence:**

The sequence of exon 1 (109bp) is shown in upper case and the flanking intronic sequences in lower case. Inclusion of this exon will lead to a frame shift.

gaaaggatagtcaacaaagactctcttgaggtggcatttgaatgaggagtgagcaagtgtcctgggcaagctctgggtcaccagcatcctcaagagatggagcaaatcaaaggcagagaccttgggaactctgatcattcttctgtttgtttgttttcgagGTTGGGTTTCTCTGTGTAACCTCGGCTGTCCAGGAACTCATTCCATAAACCAGGCTGGCCTGGAACTCAGTGATCTGCCAGCTTCTGCCTCCCAAGTGTGGGGATTAAGgttggtgacctatggctctctctctctctctttctcttgtttaaggtttatgtgtatttactgggaggtgggttgtctatgtggatctgtgtgagttggtcatgctgtgttgtacccttggaggccagaagagggtgctgcaatctctgc
